# Unmet maternal health information needs and mass media exposure for maternal health among women in the Gedeo zone, South Ethiopia

**DOI:** 10.3389/fpubh.2025.1497606

**Published:** 2025-07-23

**Authors:** Getanew Aschalew Tesfa, Addisu Getnet, Binyam Tariku Seboka

**Affiliations:** ^1^School of Public Health, College of Medicine and Health Science, Dilla University, Dilla, Ethiopia; ^2^Midwifery Department, College of Medicine and Health Science, Dilla University, Dilla, Ethiopia

**Keywords:** health information, maternal health, mass media, unmet need, women

## Abstract

**Introduction:**

The Agenda 2030 for Sustainable Development Goals (SDGs) of the United Nations emphasizes that any society’s social, political, and economic well-being depends on access to pertinent health information. To lower maternal and child mortality, it is vital to provide mothers with timely and relevant health information for informed decision-making. However, there is a limited study about unmet maternal health information needs and mass media exposure towards maternal healthcare among women in Ethiopia. Therefore, this study aimed to evaluate unmet maternal health information needs and mass media exposure among women in the Gedeo zone, South Ethiopia.

**Methods:**

A community-based cross-sectional study was conducted from February to March 2023 among 845 women who had given birth in the last 2 years before the survey. A multistage sampling technique was employed to select the study participants. Chi-square tests were used to show the relationship between categorical variables. Logistic regression was employed to identify the existence of statistically significant associations. An adjusted odds ratio (AOR) with a 95% confidence interval (CI) was used to show the strength of the association between the dependent and independent variables. A *p*-value less than 0.05 was used to declare statistical significance.

**Results:**

Of the total study participants, 72.5% (95% CI: 69.34–75.37) had high unmet maternal health information needs. Only 33.9% (95% CI: 30.80–37.19) of participants reported that they are exposed to mass media for maternal health information. Living in rural areas (AOR = 0.3, 95%CI: 0.13–0.69), primary school education (AOR = 1.6, 95%CI: 1.15–2.28), household monthly income 4,501–6,000 birr (AOR = 2.8, 95%CI: 1.72–4.64), household monthly income >6,000 birr (AOR = 3.4, 95%CI: 1.68–7.07), counseled by health extension workers (AOR = 2.0, 95%CI: 1.41–2.82), visiting health facilities (AOR = 1.5, 95%CI: 1.02–1.99), and owning mobile phones (AOR = 3.4, 95%CI: 2.01–5.75) were significantly associated with mass media exposure.

**Conclusion:**

Nearly three-quarters of the participants reported that they had high unmet maternal health information needs. Healthcare organizations, policymakers, and other governmental and non-governmental organizations should continuously work on maternal health educational programs by using different types of mass media platforms to fulfill the information needs of women.

## Introduction

Maternal health is the health of a woman during pregnancy, childbirth, and the postnatal period (1). About 260, 000 women died during and following pregnancy and childbirth in 2023 globally. Almost 92% of all maternal deaths occurred in low and lower-middle-income countries in 2023, and most could have been prevented (2). Recent data indicates that every day, almost 800 women pass away from pregnancy- and childbirth-related avoidable causes. This happens to one woman every 2 min on average, globally (3). This figure is unacceptably high. Complications during and after pregnancy and childbirth are the leading cause of death for women. About 75 % of all maternal deaths are caused by the following primary implications: infections, heavy bleeding, pregnancy-related high blood pressure, complications from pregnancy, and unsafe abortion (4).

Governmental and international organizations have committed to lowering maternal mortality globally in recognition of how significant mother and child health issues are (5). For instance, achieving good health is essential to achieving all Sustainable Development Goals (SDGs) and is a cornerstone of the 2030 Agenda for sustainable development (6, 7). Many national and international women’s health initiatives place a high premium on improving maternal and newborn health (8, 9). The main recommendation at the international and national levels for improving women’s birth outcomes is to provide effective health information (10). This can help a woman feel in charge during labor and delivery and that her wishes are respected, which can help her have a positive birth experience (11, 12). Women’s incapacity to effectively address their informational needs related to pregnancy removes their power to decide on these matters (13).

Health information serves as a vital resource that can be communicated through various media channels to effectively enhance health outcomes (8). The United Nations’ 2030 Agenda for Sustainable Development Goals underscores the importance of access to pertinent health information as a foundational element for enhancing the economic, political, and social well-being of communities (14). This access enables individuals to make informed decisions about their health, fosters greater community engagement, and ultimately contributes to stronger, more resilient societies. Thus, providing mothers with timely and relevant health information helps them to make informed decisions, which is essential in reducing pregnancy-related complications that may lead to low maternal and child mortality.

Over 80% of community homes worldwide, particularly in Sub-Saharan Africa, have inadequate access to effective, accurate, efficient, and quality mother and child health information and rely entirely on health experts and community health workers (15). Women in low and middle-income countries hardly access adequate, accurate information, the information that is appropriate to meet their needs, either in content or in presentation [in their language familiar to them during and after childbirth as the number of women dying during childbirth is still high (16)]. Given that face-to-face communication frequently involves too many human resources and only reaches a small number of individuals in broad, neglected rural areas, the mass media plays a critical role in helping health professionals reach a wider audience. The mass media serves as a crucial conduit between the people living in rural areas and important health information. Target audiences can be effectively influenced to adopt new behaviours or reminded of important information via the mass media, which includes radio and Television (17). Mass media, a form of mass communication, disseminates information, opinions, advocacy, and other forms of expression to a large audience. Television, radio, and print have all been regarded as mass media in this most general sense of the term.

Delivering health information without a deep understanding of mothers’ specific needs and preferred communication channels can fall short of meeting their fundamental requirements (18). This lack of understanding may not only leave mothers feeling unsatisfied but also increase the risk of conveying inaccurate information, which can have serious implications for their health and well-being. On the other hand, addressing these women’s interests and information needs can motivate them to accept the information and put it to better use in their lives (19).

Understanding what consumers access and tune in to health information ensures that the designed health communication messages are placed on the right channel where the target audience is looking for those messages (20). To effectively guide maternal health practitioners and to create impactful maternal health messages, it’s essential to understand the unmet needs for maternal health information, the extent of exposure to mass media, and the factors influencing these aspects. This knowledge will help us better address the needs of mothers and improve overall maternal health outcomes.

It should be emphasized that to produce effective maternity-related health information, mothers’ viewpoints must be incorporated, starting with well-designed studies. It is therefore crucial to evaluate the degree of unmet maternal health information need, and media exposure in Ethiopia at the sub-national level, which has a paramount advantage for mothers, programmers, and the community as well. Hence, this study focused on women residing in the Gedeo zone due to prior research indicating a low utilization of maternal health services (21), which influenced our choice to focus on this particular population. Additionally, there is limited evidence regarding unmet maternal health information needs and mass media exposure of Ethiopian women. Therefore, this study aimed to assess the level of unmet maternal health information needs and mass media exposure among women in the Gedeo zone, southern Ethiopia.

## Methods and materials

### Study setting

This study was done in the Gedeo zone, South Ethiopia. Gedeo is a zone in the southern region of Ethiopia and it is bordered by Oromia on the east, south and west, and it shares its northern boundary with the Sidama region. Dilla town is the headquarters of the zone, which is 359 kilometers far from Addis Ababa, Ethiopia’s capital. The zone’s geographical location is between 50,840–60,430 N latitudes and 380,080-380440E longitudes (22). Concerning healthcare infrastructure, there are four public hospitals, thirty-eight health centers, and one hundred forty-six health posts in the zone (Source: Gedeo Zone Health Bureau). Additionally, according to the zonal health office report, the total estimated population of the zone in 2021/2022 was 1,112,951. Of the total population, it is estimated that there are 239,053 reproductive-aged women and girls in the zone.

### Study design and period

The study was conducted from February to March 2023 using a community-based cross-sectional study design in the Gedeo zone, South Ethiopia.

### Population

#### Source population

All women who gave birth in the last 2 years before the study and who were residing in the Gedeo zone, South Ethiopia.

#### Study population

Selected women who gave birth in the last 2 years before the survey, residing in the selected enumeration areas of the Gedeo zone were considered as the study population.

### Eligibility criteria

#### Inclusion criteria

The population of interest for this study was all reproductive-aged women who gave birth in the last 2 years before the survey.

#### Exclusion criteria

Mothers who resided less than 6 months in the study area at the time of the survey, who were critically ill and had communication problems were not considered.

### Sample size determination

The sample size of the study was computed using a single population proportion formula using the assumption of a 95% CI with a 5% margin of error, a 50% proportion (*p* = 0.5).


$$n=\frac{{\left(Z_{a/2}\right)}^{2}p(1-p)}{d^{2}}$$

$$=(1.96)\frac{0.5(1-0.5)}{0.5^{2}}$$

=384

Where; *n* = sample size, *z* = standard normal deviation, *p* = proportion, and *d* = margin of error.

Next, we accounted for a 10% non-response rate, calculated as 384 * 10% = 38.4. This brings the adjusted total to 422.4 (384 + 38.4). Additionally, since we applied a multi-stage followed by systematic random sampling technique, a design effect is expected. In our case, with two stages, the design effect is 2. Therefore, we multiplied the sample size by two (422.4*2), resulting in a final sample size of 845.

## Sampling technique and procedure

A multistage sampling method followed by a systematic random sampling technique was used to ascertain the study subjects. First, from the total of 8 woredas (districts) and 4 city administrations of the zone, four districts and two city administrations were chosen randomly using a lottery method. Then, a total of thirty kebeles (the lower administrative unit of Ethiopia) were picked from the selected districts and city administrations randomly. Next, mothers who gave birth in the last 2 years of the study were identified from the health extension worker’s registration book (log books) in each selected kebele. And then, study participants were allocated to each selected kebele proportionally. Finally, after having a list of all eligible women in each selected kebele, 845 women were included/interviewed using a systematic random sampling technique.

Initially, 4,603 women were eligible to participate in the interview process. Then, to effectively select study subjects, we used systematic random sampling techniques. The first step was to calculate the sampling interval (K) by dividing the total eligible population of 4,603 women by our estimated sample size of 845. This calculation gave us a sampling interval of 5, meaning that every fifth woman from the list would be chosen for the study. To decide which woman would be the first participant, we employed a lottery method, ensuring a fair and unbiased selection process from the initial group.

### Study variables

#### Dependent variables

Unmet health information needs, and mass media exposure.

#### Independent variables

The independent variables of the study includes age, maternal educational level categorized as (no formal education, primary school, secondary and above), work status (not working, working), residence (urban, rural), average household monthly income categorized as (<=1,500 ETB, 1501–3,000 ETB, 3,001–4,500 ETB, 4501–6,000 ETB, >6,000 ETB), family size, own mobile phone (yes/no), having TV (yes/no), have radio (yes/no), type of health information sources, health-related decision making power categorized as (self, jointly with partner, husband alone/someone else), counseled by health extension workers (yes/no), number of children ever born, health facility visit (yes/no), place of delivery of the last child (home, health facility), antenatal care (ANC) follow-up status of the last delivery, frequency of reading newspapers/magazine (not at all, at least once a week, almost every day), frequency of listening to a radio (not at all, at least once a week, almost every day), frequency of watching TV (not at all, at least once a week, almost every day).

### Operational definition

Unmet need of maternity-related health information is defined as the mother’s unsatisfied need for adequate, timely, and understandable maternity-related health information. A total of nine questions were employed to assess unmet health information needs related to maternity. Respondents who scored below the mean (50%) on the total maternity-related health information needs questions were classified as having a “high unmet need,” while those who scored at or above the mean were classified as having a “low unmet need.”

Mass media exposure: Women’s mass media exposure to maternal healthcare information was measured by asking respondents how often they listen to a radio, read a newspaper, or watch television. All three variables had the same response options: “not at all,” “at least once per week,” and “almost every day.” These variables were then recoded into dichotomous variables, with the response options categorized as ‘not exposed’ (for women who responded not at all and ‘exposed’; combining women who responded ‘at least once a week’ and ‘almost every day’).

### Data collection procedure and quality control

Data was collected using a pretested, structured, interviewer-administered questionnaire. Initially, the tool was designed in English language. It was then translated into the local languages, Gedeoffa and Amharic, by language experts. This enabled interviews to be conducted in these local languages, which improved communication and understanding with the community. The questionnaire was adopted from prior studies (19, 23–25) and the comprehensive health information-seeking model (26). The comprehensive information seeking model originates as a combination of three concepts: the Health Belief Model, the Uses and Gratifications theory, and the model of Media Exposure and Appraisal. It is widely used to study health information-seeking behaviors. Although the model was initially developed to examine cancer-related information-seeking behaviors among patients, it has since proven applicable across various disciplines and contexts. Following this adaptation, a pretest was conducted to rigorously assess its functionality and reliability. Additionally, the content validity of the tool was meticulously evaluated by a panel of subject matter experts, ensuring that it meets the necessary standards and accurately reflects the intended concepts.

The tool has four sections. Section 1: sociodemographic information (contains 12 items); Section 2: health information sources, mass media exposure, and health-related decision-making autonomy (contains 10 items); Section 3: unmet health information need (9 items); and Section 4: access to mass media infrastructures (5 items). The consistency between the items was tested by using Cronbach’s alpha test.

A total of 10 data collectors and two supervisors were recruited for data collection. Training was given to data collectors and supervisors for two consecutive days about the objective of the study, ethical issues, and overall study procedures by the investigators. Before the actual data collection, a pretest was conducted to check the clearness of the language, appropriateness, and consistency in 5% of the total sample in the kebeles that were not selected for the study, and then all the necessary modifications to the data collection instrument were carried out. Regular meetings were held between data collectors, supervisors, and investigators to solve any challenging issues. Each questionnaire was reviewed for completeness on an everyday basis during the data collection period.

### Data processing and analysis

Data was collected using Kobo Collect (a digital data collection tool) and was exported to STATA version 14 software for analysis. Data cleaning, recoding of all required variables, and computations were done before any statistical analysis. Descriptive statistics were performed to describe the study population and to summarize the data. Categorical variables were described using frequencies, percentages, and graphs, whereas mean and standard deviation were used to summarize numerical variables. To identify the association between unmet health information needs, media exposure, and other categorical variables chi-square test was done. Furthermore, logistic regression was employed to identify factors associated with media exposure. Model fitness was checked using the Hosmer and Lemeshow goodness of fit test before running the final model. First, bi-variable analysis was done to assess the relationship of each possible independent variable with the dependent variables separately, and then variables having *p*-value <0.2 were further considered in the multivariable logistic regression analysis. Odds ratio (OR) with 95%CI and *p*-values were considered to describe the strength and direction of the association between mass media exposure and the potential predictor variables. A *p*-value less than 0.05 was used to declare the existence of statistical significance.

## Results

### Background characteristics

The study included a total of 845 women who had given birth in the previous 2 years before the study. The mean age of the participants was 29.1 (SD = 6.43). Of the total participants, 450 (53.2%) were between the ages of 25 to 34. In terms of where they lived, the majority of them, or 793 (93.7%) lived in rural areas. And 382 (45.2%) of participants had visited health facilities in the last year. Only 106 participants had the autonomy to make independent decisions regarding their health status. Whereas the majority, or 629 (62.5%) had the power to make decisions about their personal health status based on jointly with their partners. Only 107 (12.6%) participants had mobile phones and 246 (29.1%) had a radio (Table 1).

**Table 1:** Study participants’ background information.

Variables	Category	*N*	%
Age	15–24	210	24.9
	25–34	449	53.1
	35+	186	22
Residence	Urban	53	6.3
	Rural	792	93.7
Educational level	No formal education	506	59.9
	Primary school education	304	36
	Secondary and above	35	4.1
Work status	Not working	542	64.1
	Working	303	35.9
No_ of children ever born	1	127	15.0
	2–4	341	40.4
	> = 5	377	44.6
Place of delivery of the last child	Home	604	71.5
	Health facility	241	28.5
Monthly income	<=1,500	182	21.5
	1,501–3,000	214	25.3
	3,001–4,500	199	23.6
	4,501–6,000	176	20.8
	>6,000	74	8.8
Visited HF in the last year	No	464	54.9
	Yes	381	45.1
counseled by HEW in the last year	No	581	68.8
	Yes	264	31.2
Decision-making power on personal health	Self	108	12.8
	Jointly with partner	529	62.6
	Husband alone/ someone else	208	24.6
Own mobile phone	No	738	87.3
	Yes	107	12.7
Own radio	No	599	70.9
	Yes	246	29.1
Own a TV	No	801	94.8
	Yes	44	5.2

HF, health facility; HEW, health extension worker; TV, Television.

### Unmet maternal health information needs

Of the total study participants, nearly three-fourths of them, or 72.5% (95% CI: 69.34–75.37) had high unmet maternal health information needs. Besides this, unmet maternal health information needs were varied by various participants’ background characteristics. The X^2^ test also revealed that there is a significant relationship between unmet maternal health information needs and other categorical variables. Regarding their background characteristics, place of residence was significantly related to unmet health information needs (X^2^ = 5.5, *p* = 0.019), Of the total rural residents, the majority of them (73.4%) have high unmet maternal health information needs. The educational status of a woman was also highly related to maternal health information needs (X^2^ = 35.2 with *p*-value < 0.001), with increasing the educational status of a woman, unmet maternal health information needs will be decreased. Among mothers who did not attend formal education, 398 (78.7%) of have high unmet maternal health information needs and only 108 (21.3%) have low unmet needs.

Women’s autonomy in healthcare decision-making also influenced the levels of unmet maternal health information needs (X^2^ = 7.65, *p*-value = 0.022). A significant number (62.4%) of mothers who have decision-making authority over their healthcare face high unmet health information needs. Additionally, a notable 72.8% of women who participate with their partners in health decisions also experience high unmet needs. Moreover, among mothers whose husbands or someone else made decisions on their healthcare status, 76.9% of them had high unmet needs for maternal health information.

There is also a significant variability of unmet health information needs based on the availability of technological infrastructures such as mobile phones, radio, and TV. Among women who did not own a mobile phone, 75.4% had high unmet maternal health information needs, and 52.3% of women who owned a mobile phone had high unmet health information needs (X^2^ = 24.85, *p*-value < 0.001). 75.2% of participants who did not own a radio had high unmet maternal health information needs, and 65.8% of women who owned a radio reported that they had high unmet health information needs (X^2^ = 7.56, *p*-value = 0.006).

Women who visited health facilities in the previous year had a proportion of 15% lower unmet maternal health information needs than those who did not visit health facilities (X^2^ = 22.68, *p*-value < 0.001). Similarly, mothers who were counseled by health extension workers in the last year reported that they have a proportion of 16% lower unmet health information needs than those who did not get counseling from health extension workers (X^2^ = 23.62, *p*-value < 0.001). Unmet maternal health information needs were also varied by women’s antenatal care follow-up status. Among mothers who did not have an ANC follow-up history for the last child, 98% of them reported that they had high unmet needs, whereas only 55.3% of them who had ANC follow-up had high unmet needs (X^2^ = 18.9, *p*-value < 0.001) (Table 2).

**Table 2:** Relationship between unmet maternal health information need and participants’ background information.

Variables	Unmet health information need		Chi-square	*p*-value
	High *n* (%)	Low *n* (%)		
Residence			5.5	0.019
Urban	31 (58.5)	22 (41.5)		
Rural	581 (73.4)	211 (26.6)		
Educational level			35.0	<0.001
No education	398 (78.7)	108 (21.3)		
Primary	200 (65.8)	104 (34.2)		
Secondary and above	14 (40)	21 (60)		
Work status			1.89	0.169
Not working	367 (67.7)	175 (32.3)		
Working	191 (63.0)	112 (37.0)		
Counseled by HEW in the last 1 year			23.53	< 0.001
No	450 (77.5)	131 (22.5)		
Yes	162 (61.4)	102 (38.6)		
Decision-making power on personal health			7.98	0.019
Self	67 (62.0)	41 (38.0)		
Jointly with partner	385 (72.8)	144 (27.2)		
Husband alone/someone else	160 (76.9)	48 (23.1)		
Visit HFs in the previous year			22.92	< 0.001
No	367 (79.1)	97 (20.9)		
Yes	245 (64.3)	136 (35.7)		
ANC follow-up of the last birth			189.1	< 0.001
No	330 (98.5)	5 (1.5)		
Yes	282 (55.3)	228 (44.7)		
Place of delivery of the last child			179.3	< 0.001
Home	516 (85.4)	88 (14.6)		
Health facility	96 (39.8)	145 (60.2)		
Own mobile phone			24.76	< 0.001
No	556 (75.3)	182 (24.7)		
Yes	56 (52.3)	51 (47.7)		
Own radio			7.51	0.006
No	450 (75.1)	149 (24.9)		
Yes	162 (65.8)	84 (34.2)		
Have TV			4.13	0.042
No	586 (73.2)	215 (26.8)		
Yes	26 (59.1)	18 (40.9)		
Mass media exposure			19.10	< 0.001
Not Exposed	431 (77.2)	127 (22.8)		
Exposed	181 (63.1)	106 (36.9)		

### Mass media exposure for maternal healthcare information

**Figure 1:**
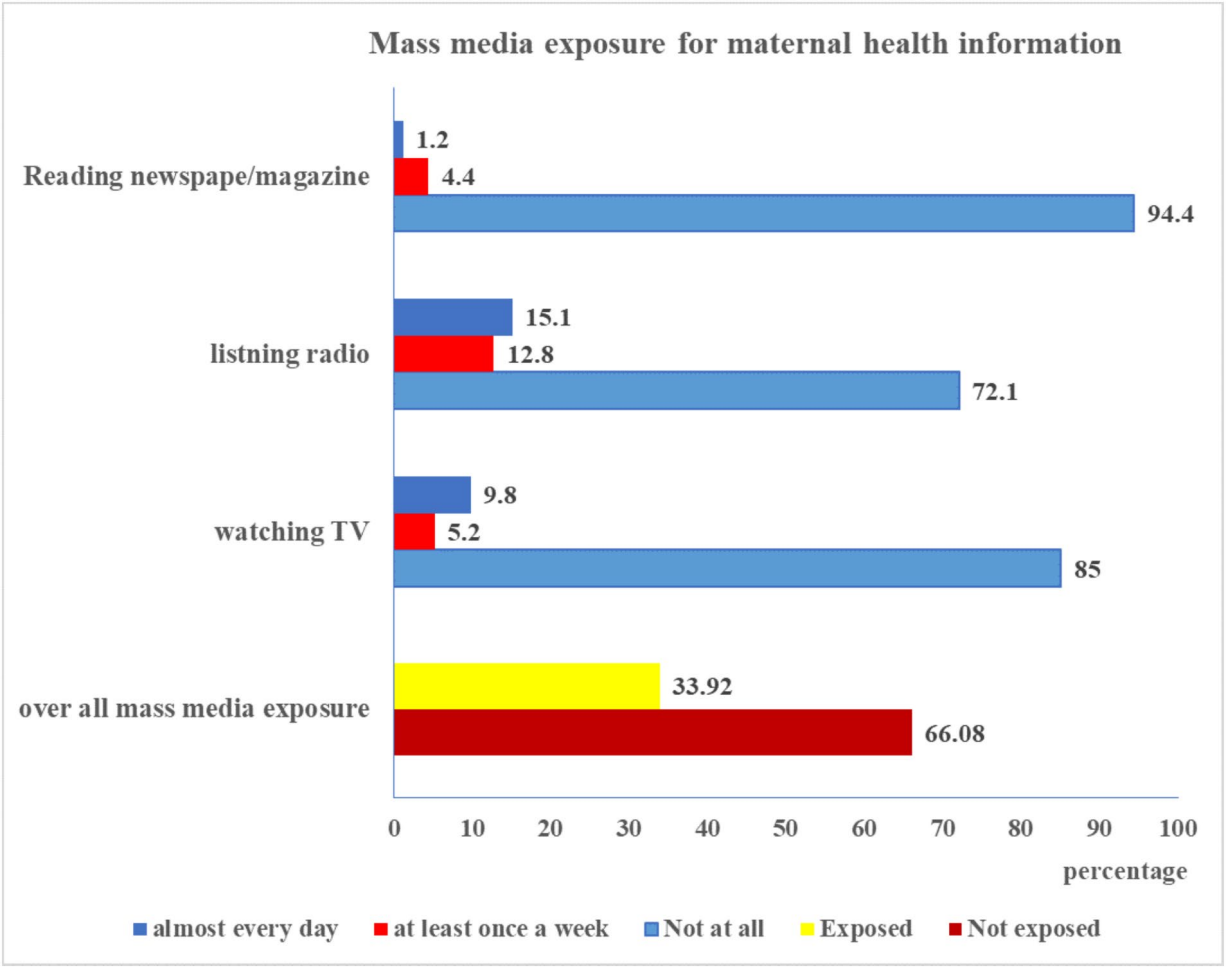
Mass media exposure among women in Gedeo zone, South Ethiopia.

Only 287 study participants or 33.9% (95% CI: 30.80–37.19) were exposed to mass media for maternal health information, as illustrated in Figure 1. Concerning the particular mass media categories, 799 study participants, or 94.4 percent never read printed health information resources (magazines or newspapers). Merely 12.8% of the total participants reported hearing about maternal health information on the radio at least once a week. Only 5.2% of mothers, on the other hand, said they watched TV at least once a week, and 9.8% said they did it nearly daily (Figure 1).

### Accessibility of mass media infrastructures

**Figure 2:**
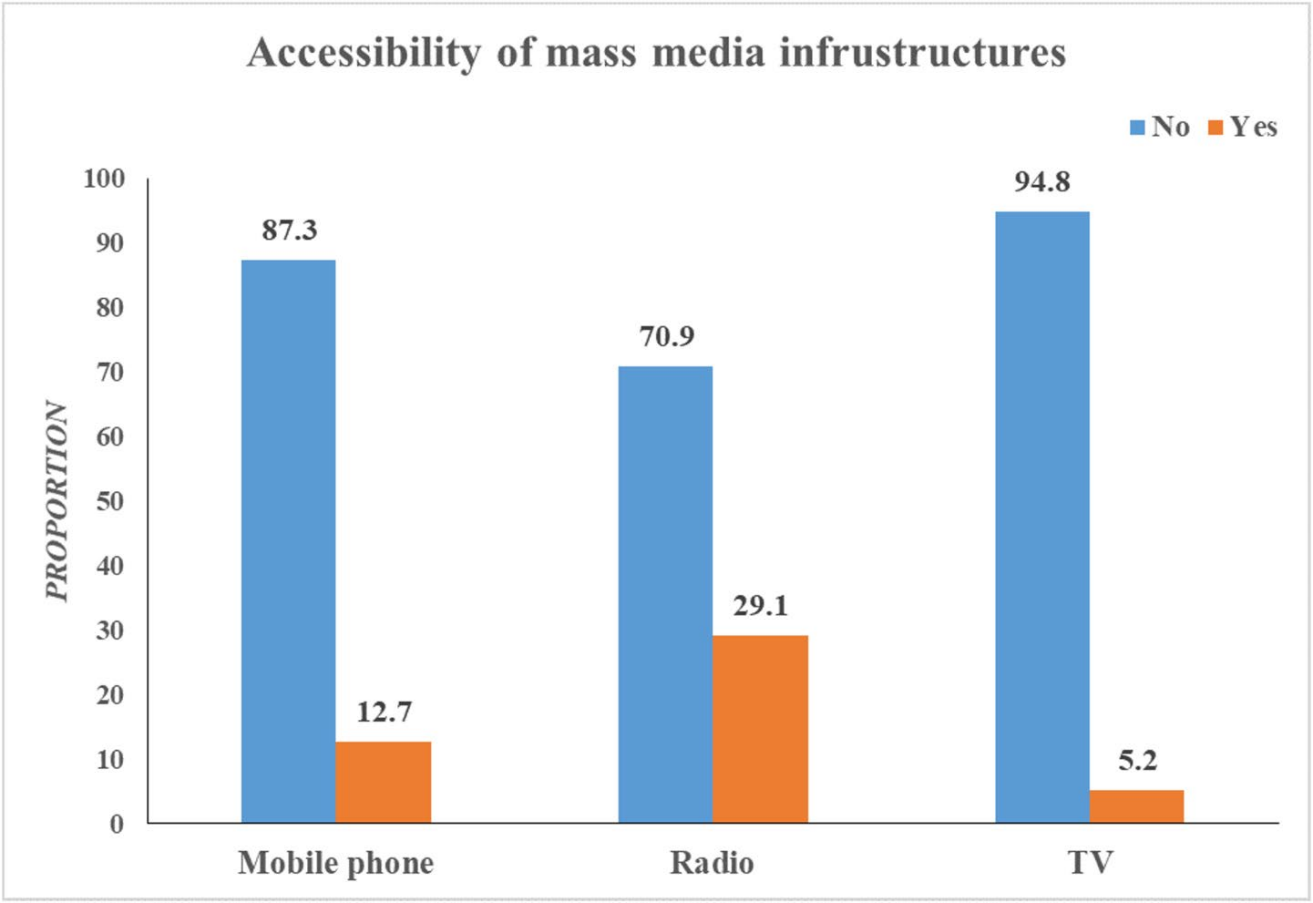
Accessibility of mass media infrastructures in the Gedeo zone, South Ethiopia.

Figure 2 below shows how women in the Gedeo zone can access mass media infrastructures. Only 107 individuals, or 12.7% of the total, indicated that they were mobile phone owners. Furthermore, 29.1%, or almost one-third, could access a radio. Just 5.2% of participants can access television (Figure 2).

### Factors associated with mass media exposure

In the bivariable logistic regression analysis; place of residence, maternal educational level, average household monthly income, counseled by health extension workers, health facility visits, decision-making power on personal health, and ownership of mobile phones were significantly associated with mass media exposure for maternal health information. However, in the multivariable analysis, only, maternal educational level, counseled by health extension workers, health facility visits, and mobile phone ownership were significantly associated with mass media exposure.

Living in a rural area had a 70% lower (AOR = 0.3, 95% CI: 0.13–0.69) chance of being exposed to mass media than living in urban settings. Additionally, this study revealed that mass media exposure was statistically associated with maternal educational status. Mothers who completed primary school education were approximately 60% more likely exposed to mass media than those mothers who did not attend any formal education (AOR = 1.6, 95% CI: 1.15–2.28). Similarly, mothers who got counseling from health extension workers had 2 times higher odds of media exposure compared to the counters who were not counseled by health extension workers (AOR = 2.0, 95% CI: 1.41–2.82).

The likelihood of being exposed to mass media was 2.8 (AOR = 2.8, 95% CI: 1.72–4.64) and 3.4 (AOR = 3.4, 95% CI: 1.68–7.07) times higher for women who were living in households with average monthly incomes of 4,501 to 6,000 ETB and higher than 6,000 ETB compared to the counterparts, respectively.

Women who owned mobile phones were three times (AOR = 3.0, 95% CI: 2.01–5.75) more likely to be exposed to mass media than those who did not own. The odds of being exposed to mass media were 1.5 (AOR = 1.5, 95% CI: 1.02–1.99) times higher among women who had a prior history of visiting health facilities compared to their counterparts (Table 3).

**Table 3:** Bi-variable and multivariable analysis of mass media exposure for maternal health information.

Variables	Mass media exposure		COR (95%CI)	AOR (95%CI)
	Unexposed *n* (%)	Exposed *n* (%)		
Place of residence				
Urban	10 (18.9)	43 (81.1)	1	1
Rural	548 (69.2)	244 (30.8)	0.1 (0.05–0.21)	0.3 (0.13–0.69)*
Educational level				
No education	382 (75.5)	124 (24.5)	1	1
Primary	163 (53.6)	141 (46.4)	2.8 (1.97–3.62)	1.6 (1.15–2.28)*
Secondary and above	13 (37.1)	22 (62.9)	5.2 (2.56–10.68)	1.3 (0.52–3.18)
Average household monthly income				
<=1,500 ETB	145 (79.7)	37 (20.3)	1	1
1,501–3,000	166 (77.6)	48 (22.4)	1.2 (0.69–1.83)	0.9 (0.52–1.46)
3,001–4,500	138 (69.4)	61 (30.6)	1.7 (1.08–2.77)	1.4 (0.87–2.34)
4,501–6,000	87 (49.4)	89 (50.6)	4.0 (2.51–6.39)	2.8 (1.72–4.64)**
>6,000	22 (29.7)	52 (70.3)	9.3 (5.01–17.14)	3.4 (1.68–7.07)**
Own mobile phone				
No	530 (71.8)	208 (28.2)	1	1
Yes	28 (26.2)	79 (73.8)	7.2 (4.55–11.41)	3.4 (2.01–5.75)**
Counseled by HEW in the last year				
No	419 (72.1)	162 (27.9)	1	1
Yes	139 (52.7)	125 (47.3)	2.3 (1.723.15)	2.0 (1.41–2.82)**
Decision-making autonomy on personal health				
Self	61 (56.5)	47 (43.5)	1	1
Jointly with partner	352 (66.5)	177 (33.5)	0.7 (0.44–1.01)	0.7 (0.42–1.10)
Husband alone/someone else	145 (69.7)	63 (30.3)	0.6 (0.35–0.92)	0.7 (0.43–1.30)
Visited health facilities				
No	337 (72.6)	127 (27.4)	1	1
Yes	221 (58.0)	160 (42.0)	1.9 (1.44–2.56)	1.5 (1.02–1.99)*

*p-value <= 0.05, **p-value <= 0.001.

## Discussion

This study attempted to assess the levels of unmet maternal health information needs and mass media exposure for maternal healthcare information among recently delivered women in the Gedeo zone, South Ethiopia. In addition to identifying the levels of unmet health information needs and mass media exposure, this study also explored the variation of unmet health information needs by various background characteristics and identified the associated factors of mass media exposure. The study revealed that the majority of the study participants, or 72.5% (95% CI: 69.34–75.37) had high unmet maternal health information needs. A literature review has shown that women in various countries often lack essential maternal health information during pregnancy, childbirth, and postpartum. Common unmet needs include guidance on nutrition, pregnancy complications, infant care, and recovery (25). In many low- and middle-income countries, cultural barriers, low literacy rates, and limited access to healthcare professionals create significant gaps in maternal health information (25).

The proportion of unmet maternal health information needs is varied by participants’ background characteristics, specifically by educational level, place of residence, decision-making power on own health, household monthly income, getting counseling by health extension workers, exposure to mass media, history of visiting health facilities, and accessibility of mass media infrastructures. Women who reside in rural areas had a higher proportion of unmet health information needs than those who live in urban areas. The differences in unmet health information needs by place of residence are probably a result of the diverse antenatal care follow-up scenarios and the country’s information sources. For instance, women may not frequently get midwives or nurses as information sources in rural areas where there are limited healthcare professionals and minimal media coverage. To reduce the high proportion of unmet health information needs, community-driven mass media campaigns by engaging professional healthcare workers may be a good alternative to educate every woman about maternal health, particularly in rural areas (27).

This study shows that unmet maternal health information needs were varied by the mothers’ educational status, with increasing the educational level of the women, unmet maternal health information needs will be decreased. Women who did not attend any formal education reported a higher proportion of unmet maternal health information needs. Education has an impact on people’s information needs and seeking behavior. As a result, educated women are more likely to have access to information on maternal health than illiterate women (28). Moreover, educated women are more likely to look for knowledge and use a range of health information sources to satisfy their desires (29).

A statistically significant relationship was identified between unmet maternal health information needs and mass media exposure. The proportion of high unmet health information needs among mass media-exposed women was 14% lower than those who had no exposure to mass media. Some evidence shows that mass media such as radio is an effective way of disseminating health information by health institutions and received by mothers. Most mothers for instance acknowledged being reminded of the child health week initiatives through radio and television adverts.

This study showed that the level of mass media exposure for maternal health information was 33.9% (95% CI: 30.80 to 37.19) among recently delivered women in the Gedeo zone, South Ethiopia. The factors associated with mass media exposure are place of residence, maternal educational level, household income, getting counseling from health extension workers, health facility visits, and accessibility of mobile phones. Place of residence was also statistically associated with mass media exposure. Women who reside in rural areas had a lower chance of being exposed to mass media. The possible explanation for this might be that geographical distance can affect people’s access to information and technology, which can affect their capacity to receive and comprehend messages. People who live in remote areas, for instance, might not have easy access to electricity, internet, TV, or other types of technology (18).

The result shows that maternal educational level is a determinant factor of mass media exposure towards maternal healthcare among recently delivered women. Supported by a study done in South Asia (30), women who attended primary school education had higher odds of mass media exposure than women who did not attend any formal education. The possible reason for this might be that educated women are more likely to have access to the media and can read or listen to information about maternal healthcare that may be broadcast on radio, TV, and in magazines or newspapers. Additionally, education has an impact on people’s information needs and seeking behavior. As a result, educated women are more likely to have access to information on maternal health than illiterate women.

Being counseled by health extension workers had a significant effect on exposure to mass media. Women who were counseled by health extension workers are more likely to be exposed to mass media compared to women who were not counseled by health extension workers. This finding is supported by the previous study done in Ethiopia (30). The possible explanation could be that health extension workers may encourage and counsel the woman to read or attend the available mass media options during her visit. Likewise, having a history of visiting health facilities had also higher odds of being exposed to mass media, women who visited health facilities were approximately two times more likely for mass media exposure than those who did not visit health facilities.

This study also revealed that household income was statistically linked to mass media exposure. The likelihood of being exposed to mass media was higher for women who were living in households with average monthly incomes of 4,501 to 6,000 ETB and higher than 6,000 ETB compared to their counterparts. Women who have high incomes have a high probability of accessing media infrastructures (30), which leads to increasing mass media exposure. Ownership of technological products such as mobile phones is a significant determinant factor of mass media exposure. Women who owned mobile phones had approximately two times higher odds of mass media exposure compared to their counterparts. Evidence shows that mobile phones have roles for accessing health messages (17, 18). The possible reason for this could be with mobile devices, people may easily find and obtain health messages whenever they want. There is a weekly health program that is scheduled in advance and is openly promoted in the mass media. As a result, through advertisements, everybody owning a mobile device will be aware of the type and timing of health-related messages to be transmitted, which gives them a chance to plan ahead and so they will not miss the program.

## Conclusion and recommendations

This study identified a high level of unmet maternal health information needs, which significantly varied by participants’ background characteristics. The study also observed that mass media exposure was significantly related to unmet health information needs. Moreover, the level of mass media exposure was low. Women’s educational status, counseling by health extension workers, and accessibility of media infrastructures such as mobile phones were associated with exposure to mass media for maternal health information. We recommend that healthcare organizations, policymakers, and other governmental and non-governmental organizations should continuously work on maternal health educational programs by using the different types of mass media platforms to fulfill the information needs of women. Improving investments in the accessibility of mass media infrastructures for mothers is also highly recommended. To effectively meet the information needs of women, appropriate, timely, and professional interventions should be devised. Moreover, collaborating with local health authorities and community stakeholders is essential for designing and implementing effective strategies to fulfill maternal health information needs.

### Limitations

This study focused on a specific geographic area where maternal health service utilization was found to be low. However, focusing on just one zone may limit the representativeness of the findings for the entire country. Therefore, we recommend that future researchers expand their studies to include multiple regions or conduct research at the national level to improve the representativeness of the results. Additionally, this study employed a quantitative approach, which was dictated by the specific objectives of our research. This focus limited our ability to address certain questions, such as evaluating women’s sanitation practices and the quality of maternal care provided. Consequently, we recommend that future studies incorporate qualitative approaches to gain a more comprehensive understanding.

## Data Availability

The raw data supporting the conclusions of this article will be made available by the authors, without undue reservation.

## References

[1] World Health Organization. (2025). Maternal health. Available online at: https://www.afro.who.int/health-topics/maternal-health (Accessed April 10, 2025).

[2] World Health Organization. (2023). Maternal mortality. Available online at: https://www.who.int/news-room/fact-sheets/detail/maternal-mortality (Accessed April 10, 2025).

[3] United Nations Population Fund. (2024). Maternal health. Available online at: https://www.unfpa.org/maternal-health (Accessed January 20, 2024).

[4] Cresswell JA Alexander M Chong MYC Link HM Pejchinovska M Gazeley U. Global and regional causes of maternal deaths 2009–20: a WHO systematic analysis. Lancet Glob Health. (2025) 13:e626–34. doi: 10.1016/S2214-109X(24)00560-6, PMID: 40064189 PMC11946934

[5] Adama AH. Preventing maternal and child mortality: upcoming WHO resolution must galvanise action to tackle the unacceptable weight of preventable deaths. Lancet Glob Health. (2024) 12:e1223–4. doi: 10.1016/S2214-109X(24)00220-1, PMID: 38782013

[6] Jenkins MG Ford JB Morris JM Roberts CL. Women's expectations and experiences of maternity care in NSW – what women highlight as most important. Women Birth. (2014) 27:214–9. doi: 10.1016/j.wombi.2014.03.002, PMID: 24746379

[7] United Nations. (2022). Ensure healthy lives and promote well-being for all at all ages. Available online at: https://sdgs.un.org/goals/goal3 (Accessed January 21, 2023).

[8] World Health Organization. (2016). World health statistics 2016: monitoring health for the SDGs. Available online at: https://reliefweb.int/report/world/world-health-statistics-2016-monitoring-health-sdgs (Accessed January 21 2023).

[9] Jolivet RR Gausman J Kapoor N Langer A Sharma J Semrau KEA. Operationalizing respectful maternity care at the healthcare provider level: a systematic scoping review. Reprod Health. (2021) 18:194. doi: 10.1186/s12978-021-01241-5, PMID: 34598705 PMC8485458

[10] United Nations. (2023). Ensure inclusive and equitable quality education and promote lifelong learning opportunities for all. Available online at: https://sdgs.un.org/goals/goal4 (Accessed January 21, 2023).

[11] Ames HM Glenton C Lewin S. Parents' and informal caregivers' views and experiences of communication about routine childhood vaccination: a synthesis of qualitative evidence. Cochrane Database Syst Rev. (2017) 2:Cd011787. doi: 10.1002/14651858.CD011787.pub2 28169420 PMC5461870

[12] Kassim M. A qualitative study of the maternal health information-seeking behaviour of women of reproductive age in Mpwapwa district, Tanzania. Health Info Libr J. (2021) 38:182–93. doi: 10.1111/hir.12329, PMID: 33052617 PMC8518957

[13] Javanmardi M Noroozi M Mostafavi F Ashrafi-Rizi M. Exploring women's health information needs during pregnancy: a qualitative study. J Family Reprod Health. (2020) 14:252–8. doi: 10.18502/jfrh.v14i4.5209, PMID: 34054997 PMC8144486

[14] Temmerman M Khosla R Laski L Mathews Z Say L. Women’s health priorities and interventions. Br Med J. (2015) 351:h4147. doi: 10.1136/bmj.h4147, PMID: 26371215

[15] Tsawe M Susuman AS. Determinants of access to and use of maternal health care services in the eastern cape, South Africa: a quantitative and qualitative investigation. BMC Res Notes. (2014) 7:723. doi: 10.1186/1756-0500-7-723 25315012 PMC4203863

[16] Mwangakala HA. Accessibility of maternal health information and its influence on maternal health preferences in rural Tanzania: a case study of Chamwino District. South Afr J Inform Manag. (2021) 23:1–9. doi: 10.4102/sajim.v23i1.1353

[17] Naveena N. Importance of mass Media in Communicating Health Messages: an analysis. IOSR J Human Soc Sci. (2015) 20:36–41. doi: 10.9790/0837-20253641

[18] Aliyi BDY Deressa A Debella A Birhanu A Gamachu M Eyeberu A. Demand of and access to health messages through mass Media in the Rural Community of eastern Ethiopia: a mixed method study. Risk Manag Healthc Policy. (2023) 16:1859–74. doi: 10.2147/RMHP.S429712 37719689 PMC10503334

[19] Lu Y Barrett LA Lin RZ Amith M Tao C He Z. Understanding information needs and barriers to accessing health information across all stages of pregnancy: systematic review. JMIR Pediatr Parent. (2022) 5:e32235. doi: 10.2196/32235, PMID: 35188477 PMC8902674

[20] Stellefson M Paige SR Chaney BH Chaney JD. Evolving role of social Media in Health Promotion: updated responsibilities for health education specialists. Int J Environ Res Public Health. (2020) 17:1153. doi: 10.3390/ijerph17041153, PMID: 32059561 PMC7068576

[21] Muluneh AA Kassa ZY Mamo ZB Hadra N. The utilization of antenatal care and associated factors in Gedeo zone, southern Ethiopia: utilization of antenatal care in Gedeo zone, southern Ethiopia. Ethiop J Reprod Health. (2021) 13:458. doi: 10.69614/ejrh.v13i01.458

[22] Wikipedia. (2021). Gedeo Zone. Available online at: https://en.wikipedia.org/wiki/Gedeo_Zone [Accessed February 28, 2022].

[23] Wolvaardt L. A descriptive study of the health information needs of Kenyan women in the first 6 weeks postpartum. BMC Pregnancy Childbirth. (2017). doi: 10.1186/s12884-017-1576-1, PMID: 29145804 PMC5691856

[24] Kamali S Ahmadian L Khajouei R Bahaadinbeigy K. Health information needs of pregnant women: information sources, motives and barriers. Health Inf Libr J. (2018). doi: 10.1111/hir.12200 29131537

[25] Mulauzi F. Maternal health information needs of women: a survey of literature. J Lexicography Terminol. (2018) 2:57–82.

[26] Johnson JD Meischke H. A comprehensive model of cancer-related information seeking applied to magazines. Hum Commun Res. (1993) 19:343–67. doi: 10.1111/j.1468-2958.1993.tb00305.x

[27] Zamawe COF Banda M Dube AN. The impact of a community driven mass media campaign on the utilisation of maternal health care services in rural Malawi. BMC Pregnancy Childbirth. (2016) 16:21. doi: 10.1186/s12884-016-0816-0 26819242 PMC4730729

[28] Irshad Ahmad Reshi DTS Dar SA. Women's access to education and its impact on their empowerment: a comprehensive review. Morfai J. (2022) 1:446–50. doi: 10.54443/morfai.v1i2.760

[29] Fatema K Lariscy JT. Mass media exposure and maternal healthcare utilization in South Asia. SSM-Population Health. (2020) 11:100614. doi: 10.1016/j.ssmph.2020.100614, PMID: 32596437 PMC7306581

[30] Gashu KD. Factors associated with women's exposure to mass media for health care information in Ethiopia. A case-control study. Clin Epidemiol Glob Health. (2021) 12:100833. doi: 10.1016/j.cegh.2021.100833, PMID: 40630313

